# Simultaneous detection of DNA variation and methylation at HLA class II locus and immune gene promoters using targeted SureSelect Methyl-Sequencing

**DOI:** 10.3389/fimmu.2023.1251772

**Published:** 2023-08-24

**Authors:** Maria Kalomoiri, Chandana Rao Prakash, Sonja Lagström, Kai Hauschulz, Ewoud Ewing, Klementy Shchetynsky, Lara Kular, Maria Needhamsen, Maja Jagodic

**Affiliations:** ^1^ Department of Clinical Neuroscience, Center for Molecular Medicine, Karolinska Institutet, Stockholm, Sweden; ^2^ Diagnostics and Genomics Group, Agilent Technologies Sweden AB, Sundbyberg, Sweden; ^3^ Diagnostics and Genomics Group, Agilent Technologies Deutschland GmbH, Waldbronn, Germany

**Keywords:** HLA/MHC, DNA variance, DNA methylation, immune regulation, antigen presentation

## Abstract

The Human Leukocyte Antigen (HLA) locus associates with a variety of complex diseases, particularly autoimmune and inflammatory conditions. The HLA-DR15 haplotype, for example, confers the major risk for developing Multiple Sclerosis in Caucasians, pinpointing an important role in the etiology of this chronic inflammatory disease of the central nervous system. In addition to the protein-coding variants that shape the functional HLA-antigen-T cell interaction, recent studies suggest that the levels of HLA molecule expression, that are epigenetically controlled, also play a role in disease development. However, deciphering the exact molecular mechanisms of the HLA association has been hampered by the tremendous genetic complexity of the locus and a lack of robust approaches to investigate it. Here, we developed a method to specifically enrich the genomic DNA from the HLA class II locus (chr6:32,426,802-34,167,129) and proximal promoters of 2,157 immune-relevant genes, utilizing the Agilent RNA-based SureSelect Methyl-Seq Capture related method, followed by sequencing to detect genetic and epigenetic variation. We demonstrated successful simultaneous detection of the genetic variation and quantification of DNA methylation levels in HLA locus. Moreover, by the detection of differentially methylated positions in promoters of immune-related genes, we identified relevant pathways following stimulation of cells. Taken together, we present a method that can be utilized to study the interplay between genetic variance and epigenetic regulation in the HLA class II region, potentially, in a wide disease context.

## Introduction

The Major Histocompatibility Complex (MHC) known as the Human Leukocyte Antigen (HLA) locus in humans encodes genes involved in antigen presentation and activation of the adaptive immune system, and stands as one of the most polymorphic loci in the human genome (1). For example, the *HLA-DRB1* gene alone encodes nearly 2,500 known alleles (2). The rate of polymorphisms is particularly high in the sequence encoding the peptide-binding groove of the HLA complexes shaping the specificity and response of the T cell repertoire. In addition, the locus is characterized by strong linkage disequilibrium (LD), non-random associations of specific alleles at different loci within a haplotype block, and specific haplotypes have emerged in different ethnic groups (3). The heterozygote advantage and the frequency selection theories have been proposed to explain the maintenance of this high rate of polymorphisms (3, 4).

Variations in the HLA locus predispose for a myriad of diseases (5, 6) including ankylosing spondylitis (AS), rheumatoid arthritis (RA), multiple sclerosis (MS), systemic lupus erythematosus (SLE), but also schizophrenia, Parkinson and Alzheimer’s disease that are not considered to be typical immune-related diseases. More specifically, distinct haplotypes and genes in the HLA class II are among the strongest risk factors for developing different immune-mediated diseases. For example, RA has been associated with the DR4 haplotype, specifically with HLA-DRB1*04:01, *04:04 and *04:08 alleles in Caucasians (7–9). The HLA-DRB1*01, *04 and *07 alleles have been shown to contribute, in addition to the major B27 class I risk, to the risk of developing AS (10, 11). Increased susceptibility to SLE has been linked with the DR3 and DR15 haplotypes, with the predisposing alleles being the HLA-DRB1*03:01 and *15:01 in Caucasians (12). The DR15 haplotype is also associated with the development of MS and narcolepsy (13–16). More precisely, the DRB5*01:01-DRB1*15:01 allele confers 3-fold higher risk for the development of MS in individuals of north European origin (13–15). Additional alleles such as HLA-DRB1*03:01, *13:01 and *08:01 also associate with the risk of developing MS, although with a lower magnitude compared to *15:01, and protective alleles exist in class I locus (14). Importantly, the disease risk conferred by the HLA locus variants is further drastically increased in interaction with lifestyle factors, such as smoking, infection and vitamin D deficiency (17, 18).

In addition to changes in the amino acid composition of the HLA molecules, expression levels of the HLA class II genes have also been suggested to be of importance for the risk of developing disease (19–22). In MS, this effect was attributed to epigenetic changes in DNA methylation (23), i.e., the addition of a methyl-group in the 5-carbon of cytosines in CpG dinucleotides (24). Mediation of the HLA and lifestyle factors *via* DNA methylation in the HLA locus has also been demonstrated in RA (25, 26) and suggested in other immune-mediated diseases (27–31).

The role of HLA in immunity, susceptibility to disease and solid organ transplantation has rendered sequencing of the locus of the utmost importance both in clinical practice, and the identification of risk variants that predispose for the different diseases. Over the past decades, several genotyping strategies ranging from serological test (32), Sanger sequencing to more recent sequence-specific oligonucleotide-based typing (33) have contributed to unraveling the sequence polymorphism in the HLA locus. Yet, these targeted approaches often focus on the polymorphic exon 2 of genes and do not enable wide coverage of the HLA class II region. On the other hand, conventional sequencing strategies of the HLA locus showed limited read length or sequencing errors for the imputation of ambiguous genotypes (34–36). Overall, the genetic analysis of HLA remains challenging due to similarity across the genes and alleles, homopolymeric stretches in intragenic regions and the existence of many and haplotype-specific pseudogenes (37). These caveats have led to limited knowledge regarding the molecular contribution of each allele in the susceptibility of diseases (2), despite robust associations with the locus being known for decades. To address these limitations and enable investigation of the class II locus, we developed a targeted-capture approach based on the Agilent SureSelect Methyl-Seq platform for simultaneous investigation of genetic and epigenetic variations of the HLA class II locus.

## Materials and methods

### Probe design

**Figure 1:**
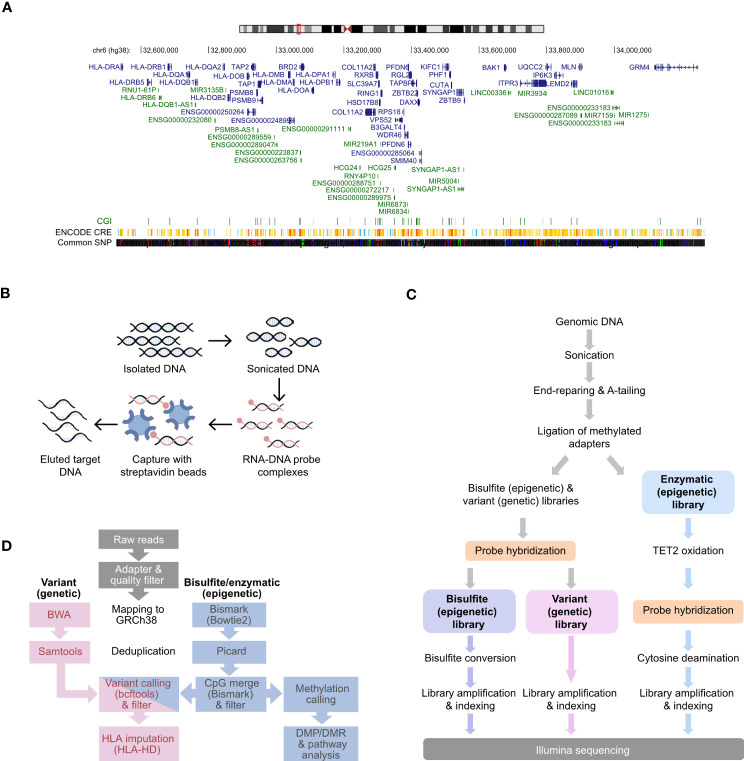
Design and workflow for simultaneous interrogation of genetic variation and DNA methylation at HLA class II locus and immune gene promoters. **(A)** Chromosome representation of the targeted HLA class II locus. Genes are depicted in blue, while pseudogenes and non-coding RNAs are represented in green. **(B)** Illustration of DNA fragment capture using biotin-labelled RNA probes based on the Agilent SureSelect Methyl-Seq Capture method. **(C)** Workflow for the parallel preparation of variant and methylation libraries adapted from SureSelect Methyl-Seq. **(D)** Bioinformatic pipeline used for the analysis of the variant and methylation libraries.

RNA probes were designed against the entire HLA class II locus sequence (GRCh38 coordinates: chr6:32,426,802-34,167,129:1) retrieved from the UCSC (GENCODE version 38lift37) genome browser (**Figure 1A**) with a density of 3X probe tiling, i.e., each base of a target region was on average covered by three overlapping probes (**Supplementary Figure 1A**). The size of each probe is 120 nucleotides and tolerate mismatches and even smaller insertions/deletions. Probes were also designed against proximal promoters of 2,157 immune-related genes (**Supplementary Table 1**) selected based on six publicly available transcriptomic data (38–42) from the monocytic/macrophage THP-1 cell line. We also utilized Hi-C data to include the interactions of the HLA with other genes using public database for THP-1 cells (43) (**Supplementary Table 2**). Genes included in the final selection displayed transcriptional changes after inflammatory stimulation in at least three independent studies, complemented by additional genes from commercial myeloid panel genes (Nanostring) (**Supplementary Table 2**). These non-HLA probes were designed based on promoter IDs obtained from the Eukaryotic Promoter Database (EPDnew released 2019, assembly GRCh38/hg38, https://epd.epfl.ch//index.php) with a 1-3X density. To improve the capture of regions with high GC%, we applied a so-called probe boosting (**Supplementary Figure 1**), i.e., a dynamic process where each designed probe is replicated up to 16 times based on the GC% of a region. For the HLA sub-design masking strategy, we selected the Least Stringent option, (https://earray.chem.agilent.com/suredesign/help/Impact_of_parameters_on_probe_selection.htm). This approach allowed for the identification of repetitive elements (such as DNA transposons, LTR, non LTR-retrotransposons, LINE and SINE elements) by all three masking tools (RepeatMasker, WindowMasker, and the Duke Uniqueness 35 track) and the subsequent masking from the probe design. Of note, the pseudogenes of the HLA locus were not masked and the probe design enables their capture. The final panel design (Design ID: S3381502) comprised 120,990 probes covering 2,223 kilobase (kb), of which 65,384 probes cover 1,621 kb of the HLA class II locus, while 55,606 probes cover 602 kb of promoter sequences (**Figure 1A**; **Table 1**).

**Table 1:** Characteristics of the probe-based DNA capture design.

	HLA class II locus	Immune gene promoters	Total
Number of probes	65,384	55,606	120,990
Size of capture (kb)	1,621	602	2,223
Probe density (tiling)	3X	< 3X	< 3X
Number of genes	81	2157	2238

kb, kilobase.

### Library preparation

A total of 3.3 μg of genomic DNA was diluted in Low TE Buffer (10 mM Tris-HCl pH 8.0, 0.1 mM EDTA) in microTUBE-50 AFA Fiber Screw-Cap (Covaris) for fragmentation on Covaris M220. The DNA was sheared at 20°C for 160 seconds and 200 cycles per burst (Covaris M220) to achieve 250 bp-long fragmentation (**Figure 1B**). The quality of DNA was assessed using the Agilent 1000 DNA kit (Agilent Technologies) in a 2100 Bioanalyzer (Agilent Technologies).

The libraries were prepared using the SureSelect^XT^ Methyl-Seq kit (Agilent, part# G9651B) and beads from the Agencourt AMPure XP kit (Beckman Coulter) were used for purification of the DNA fragments, followed by end-repairing and A-tailing (**Figure 1B**). After ligation of the methylated adapters the samples were split into two reactions in a 2:1 volume ratio in order to accomodate paralelle Bisulfite (BS) and Enzymatic (EM) conversion. Two different library preparations followed: (i) BS-Seq & Variant Library and (ii) EM-Seq Library (**Figure 1C**). For the Bisulfite Conversion & Variant Library the hybridization of the samples was performed using the Custom Probe Design (Design ID: S3381502) for 16 hours at 65°C. After the hybridization, capture of the DNA-RNA formed complexes was performed using Dynabeads MyOne Streptavidin T1 beads (Thermo Fischer Scientific) under 30 min shaking at 1800 rpm on a mixer. In the last washing step, the 200 μl sample was split into two equal aliquots for variant genomic detection and BS methylation analysis methylation analysis.

For the construction of the BS Converted library, the DNA was eluted in 20 μl of 0.1 M NaOH and processed immediately using the EZ-DNA Methylation kit (Zymo Research). BS conversion lasted 150 minutes at 64°C and the BS converted DNA was eluted in a 20 μl final volume. Subsequently, the library was amplified using the SureSelect^XT^ Methyl reagent kit for 8 cycles. For the construction of the Variant Library, 30 μl of nuclease-free water was added to the beads, the DNA was amplified using the Herculase II Fusion DNA Polymerase (Agilent Technologies) for 8 PCR cycles and the libraries were cleaned using Agencourt AMPure XP beads.

For the construction of the EM Converted library, the DNA was oxidized using the NEB Next Enzymatic Methyl-seq Conversion Module (New England BioLabs). More specifically, the DNA was diluted in a 28 μL volume and incubated with the oxidation enzymes for 1h at 37°C in a thermocycler. Enzymes were inactivated using the Stop Reagent according to the manufacturer’s instructions and the converted DNA was purified using the Agencourt AMPure XP beads. The DNA was hybridized with the custom-made probes (Design ID: S3381502) for 16 hours at 65°C. The same hybridization and capture steps were followed as mentioned previously and the DNA was eluted in 20 μl of 0.1 M NaOH. Deamination of the cytosines was performed for 3 hours at 37°C and then the samples were cleaned with AMPure XP beads. The library was amplified for 8 cycles and indexed.

The quality and quantity of the libraries were assessed using the BioAnalyzer High Sensitivity DNA Kit (Agilent Technologies) before and after pooling in equal amounts (**Supplementary Figure 2**) prior to sequencing with an Illumina NovaSeq sequencer and a 2x150bp protocol.

### Cell stimulation and DNA extraction

The THP-1 acute monocytic leukemic cell line (ATCC: The Global Bioresource Center) was expanded in complete RPMI medium composed of 10% Fetal Bovine Serum, supplemented with 1% Penicillin-Streptomycin (all from Merck KGaA) with the addition of L-glutamine, sodium pyruvate and 2-mercaptoethanol (1.7 mM, 0.87 mM and 0.02 mM, respectively). Cells were plated in a 6-well plate in complete RPMI medium and stimulated with 20 ng/ml of interferon-gamma (IFNγ) and/or 5 ng/ml lipopolysaccharide (LPS) for 24 hours. Following stimulation, cells were centrifuged at 350 g for 5 min, lysed in the RLT plus buffer (Qiagen) and kept at -80C until further use. Genomic DNA was extracted using the DNA/RNA Mini Kit (Qiagen) according to manufacturer’s instructions. The DNA was eluted in Elution Buffer (10 mM Tris-Cl, pH 8.5) and the DNA concentration was measured using the Qubit dsDNA Broad Range kit (Thermo Fischer Scientific). The purity of the extracted DNA was verified using the QIAexpert spectrophotometer (Qiagen), considering the OD260/280 absorbance ratio.

### Bioinformatic analysis

All analysis in R was conducted in version 4.0.3 (44). The sequencing data were deposited in the Gene Expression Omnibus (GEO) with accession number GSE238121.

#### Variant libraries

Reads from the variant libraries were filtered for adapter and quality using Agilent Genomics NextGen Toolkit (AGeNT) software and subsequently mapped to GRCh38 using Burrows-Wheeler Aligner (BWA), version 0.7.15 (45). BAM files were deduplicated using the markdup function of Samtools, version 1.9 (46) and overlap with the designed probes were assessed using the intersection function of BEDTools, version 2.27.1 (47). Variant calling was conducted using the mpileup, call, filter and query functions of bcftools, version 1.14 (46). HLA haplotypes were called from filtered fastq files using HLA-HD version 1.7.0 (**Figure 1D**) (48).

#### DNA methylation libraries

Reads from the DNA methylation libraries were processed and mapped to GRCh38 using the nf-core/methylseq version 1.5 pipeline with Trim Galore version 0.6.4 (github.com/FelixKrueger/TrimGalore), Bismark version 0.22.3 (49), BWA-meth version 0.2.2 (arxiv.org/abs/1401.1129) and Picard MarkDuplicates version 1.5 (broadinstitute.github.io/*picard*). Deduplicated coverage files were subsequently CpG merged using the coverage2cytosine function of Bismark version 0.23.1 (49), filtered for 10X coverage and combined using the bsseq bioconductor package version 1.26.0 (50). Methylation differences between S3 and S4 (BS libraries) and S5 and S6 (EM-seq libraries) were estimated by subtracting methylation values between the samples of interest. CpGs were consider as differentially methylated positions (DMPs) if the |methylation difference| > 0.1 and as differentially methylated regions (DMRs) if at least two consecutive CpGs were classified as DMPs (**Figure 1D**).

#### Plotting

Manhattan plots were generated with the qqman R package version 0.1.8 (51). Genetic variance was visualized using the Gviz Bioconductor package version 1.34.1 (52) and the VariantAnnotation package version 1.36.0 (53) was used for reading and processing of vcf files generated by bcftools, version 1.14 (46) as described above (**Figure 1D**).

### Gene ontology analysis

The genes closest to CpGs that displayed methylation change > 5% between the investigated conditions with the same direction of the effect in BS-seq and EM-seq analysis, were selected for downstream analysis. Different networks with the different genes were created using STRING and community clustering with 10 different clusters was performed (54). The pathways were imported to Cytoscape and were graphically segregated depicting the differentially methylated positions in each network, applying Gene Ontology analysis through BiNGO (55, 56). GO overrepresentation analysis (https://www.webgestalt.org/) was performed using default parameters and the genes targeted with custom designed probes as a background. Representative GO terms with significant FDR and adj.p-value < 0.05 were graphically depicted in the pathway analysis.

## Results

### Targeted design of the HLA class II region and immune gene promoters capture and experimental strategy

We developed a probe-based DNA capture approach to investigate the genetic variation and DNA methylation in the HLA class II locus and proximal promoters of 2,157 immune genes (**Supplementary Table 1**). The HLA class II genes and the extended class II region (**Figure 1A**, hg38, chr6:32,426,802-34,167,129:1) were covered using the density of 3X probe tiling, which provided the largest in-silico coverage (96.66%) of the target locus in comparison to higher probe tiling, i.e.4X (96.62%) and 5X (96.56). The selection of the additional 2,157 immune genes relevant for myeloid and antigen presenting cell function was based on the myeloid panel genes from Nanostring as well as genes found to be affected by inflammatory stimulation of THP-1 myeloid cell line (**Supplementary Table 2**). The genes that displayed differential expression following stimulation in several independent RNA-sequencing studies were prioritized (38–42). Each proximal promoter (average size between 100-200 bp) was covered according to regional specific parameters yielding the density of 1-3X probe tiling. The final design of the capture probes (Design ID: S3381502) comprised 120,990 probes covering 2,223 kb, of which 65,384 (54%) cover 1,621 kb of the HLA class II locus, while 55,606 (46%) probes cover 602 kb of promoter sequences (**Table 1**).

We adapted the Agilent SureSelect Methyl-Seq platform (G7530-90002, Version E0, April 2018) (**Figure 1B**) (57, 58) for simultaneous DNA and methylome analysis producing a Variant Library (DNA-seq) and Bisulfite Conversion Library (BS-seq), respectively. Additionally, we tested the compatibility of the protocol for methylome analysis using enzymatic reaction by generating an Enzymatic Methylation Library (EM-seq) (59), which is becoming increasingly popular due to less degradation of the DNA compared to BS (**Figure 1C**).

We addressed the performance of the capture probe design using the human THP-1 immortalized monocytic cell line commonly used to study monocyte/macrophage differentiation and function. Following stimulation with LPS and IFNγ, THP-1 cells upregulate expression of HLA class II genes, co-stimulatory molecules and proinflammatory cytokines (60, 61), thus providing a suitable model for the study of APC cell functions. In addition, THP-1 cells are heterozygous for the HLA class II locus, carrying the HLA-DR1 and HLA-DR15 haplotypes (62), allowing us to test the effect of haplotype sequence variation on the efficiency of the DNA capture. The cells were stimulated with IFNγ/LPS or IFNγ only for 24 hours. Three libraries for each sample (six libraries in total, hereafter referred to as: DNA-IFNγ/LPS, BS-IFNγ/LPS and EM-IFNγ/LPS and DNA-IFNγ, BS-IFNγ, EM-IFNγ) were prepared, amplified, indexed and pooled for sequencing with an average of 4.1 Gb reads per sample and 1000X coverage.

### The designed array enriches for genomic regions of interest and is highly reproducible between replicates

DNA sequencing reads from IFNγ/LPS- and IFNγ-derived samples, which have identical genomes and thus represent technical replicates, were quality and adapter trimmed and subsequently mapped to the human reference genome hg38 (**Figure 1D**). Noticeably, approximately 42% (15,771/37,336 and 15,622/37,0700) of the reads were filtered in the deduplication step for both samples. After deduplication, 99.2% and 99.4% of the reads mapped with an average insert size of 275.7 and 287.3, respectively, and an average read quality for both samples of 33.1, indicating good reliability of the sequencing data.

**Figure 2:**
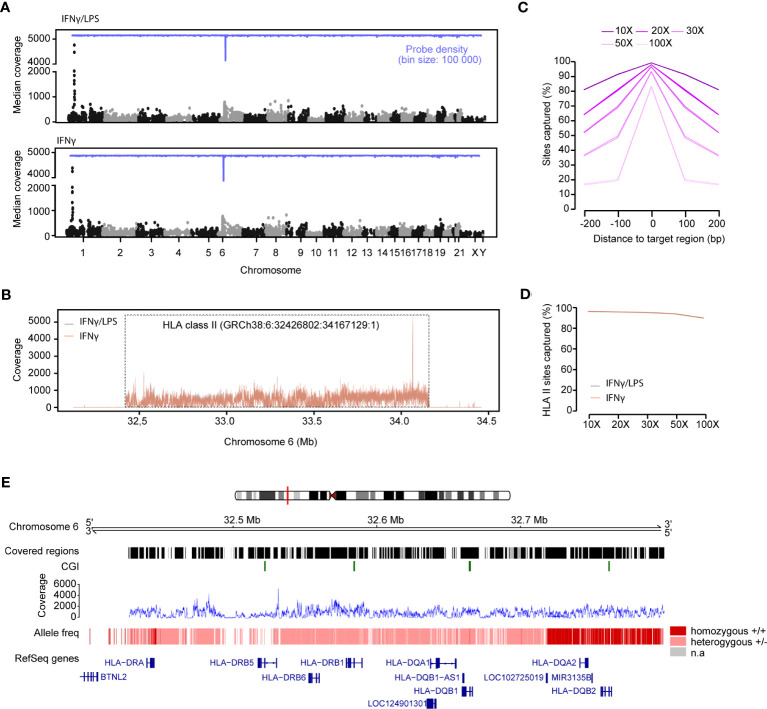
Capture efficiency and variant calling of the Variant libraries. **(A)** Manhattan plots of the median coverage in the variant libraries from DNA from THP-1 cells treated with IFNg/LPS (top) and IFNg (bottom). The probe density is depicted across different chromosomes in blue. **(B)** Mean read coverage across the HLA class II locus on chromosome 6 (GRCh38:6: 32,426,802-34,167,129:1). **(C)** Number of sites captured with different coverages (10X, 20X, 30X, 50X, 100X) across all the targeted and adjacent sequences. **(D)** Number of sites that were captured in the HLA class II region in the IFNg/LPS- and IFNg-treated samples. **(E)** Estimation of the allele frequency for the variant calling of the HLA genes in the HLA class II region. The homozygous alleles are depicted with dark red and the heterozygous alleles with light pink. Regions that were of low coverage are illustrated by gaps. Mean coverage at the HLA class II locus for both samples is depicted in blue. The RefSeq genes are depicted in blue.

Next, we assessed coverage across the 4,805 captured regions in DNA-IFNγ/LPS and DNA-IFNγ samples after applying a coverage filter of 10X (**Figure 2A**). Overall, 98.7% (4,744 of 4,805) and 98.5% (4,734 of 4,805) of the designed regions were successfully captured for each sample. We estimated a median coverage of 122X (range 11X - 4759X) across the genomic regions with little variation between the samples (**Table 2**). A locus on chromosome 1 (hg38, chr1: 40,691,614-40,692,158) displayed surprisingly high coverage in both samples (**Figure 2A**). As expected, the extended HLA class II region (h38, chr6:32,426,802-34,167,129:1) had a larger median coverage of 202X (range 12X-789X) (**Table 2**; **Figure 2B**).

**Table 2:** Coverage of the regions captured using DNA-sequencing.

	IFNγ/LPS	IFNγ
All regions (n = 4,805)		
n regions (%)	4,744 (98.7)	4,734 (98.5)
Median coverage (range)	125 (11-4759)	119 (11-4384)
HLA class II locus (n = 917)		
n regions (%)	910 (99.2)	911 (99.3)
Median coverage (range)	203 (12-789)	201 (12-767)

n, number; %, percentage.

On average, 99.2% of all the sites were covered with at least 10X, 96.55% with > 30X and 83.2% with > 100X, with little variation among the replicates (**Figure 2C**; **Supplementary Table 3**). Noticeably, sites located upstream and downstream of the target regions were also captured (**Figure 2C**), with for example ~69% and 52% of the sites within ± 100 bp and 200 bp windows proximal to the targeted region, respectively, being covered with > 30X. Again, the coverage at the extended HLA class II region was higher, as more 99.7%, 98.7% and 93.3% of the sites) were covered with than 10X, 30X and 100X, respectively (**Figure 2D**; **Supplementary Table 3**).

Taken together, the data shows that the designed probes successfully capture the genomic regions of interest, and that the data are reproducible between technical replicates and provide sufficient coverage for downstream analysis such as variant calling.

### The DNA capture array allows for variant calling and high-resolution HLA typing

We next called genotypes and inferred HLA haplotypes from the captured regions using bcftools (46) and HLA-HD (48), the latter method has previously been benchmarked for its high accuracy of HLA class II typing (63, 64). Genotypes could be called for 96.8% (2,154,496/2,221,666) and 96.6% (2,151,062/2,221,666) of the sites, respectively in each sample, at a minimum coverage of 30X. Variance, called as alternative alleles compared to the hg38 reference, was detected at 10,025 and 10,030 individual sites, respectively, the vast majority (98%, 9,822) of the variants overlapping between replicates.

As previously mentioned, the THP-1 cell line is heterozygous for the HLA class II locus, carrying both the HLA-DR1 and the HLA-DR15 haplotypes. Consistent with this, we called heterozygous genotypes within *HLA-DRB1* as well as -*DRB6*, -*DQA1*, -*DQB1* loci, whereas *HLA-DQA2*, -*DQB2* and *HLA-DRA* were homozygous based on the bcftools-derived variants (**Figure 2E**). HLA-specific typing based on the HLA-HD algorithm and dictionary, which allows for up to 6-digit calling, confirmed heterozygosity at *HLA-DRB1* (*01:01:01/*15:01:01), *HLA-DQA1* (*01:01:01/*01:02:01), *HLA-DQB1* (*05:01:01/*06:02:01) and *HLA-DRB6* (*01:01/*01:02) haplotypes (alleles). Furthermore, HLA-DMB (*01:01:01/01:03:01) and HLA-DOA (*01:01:02/*01:01:01), HLA-DPA1 (*01:03:01/02:02:02), HLA-DPB1 (*04:02:01/02:01:02) were typed as heterozygous, whereas HLA-DMA*01:01:01, HLA-DOB*01:01:01, HLA-DRA*01:01:01, HLA-DRB5*01:01:01 and HLA-DRB9*01:01 were homozygous, as expected (**Table 3**). *HLA-DRB2*, -*3*, -*4*, -*7* and -*8* pseudogenes were not typed. Of note, the HLA-HD-based typing conducted on quality-processed and adapter-trimmed fastq files yielded consistent results before and after deduplication.

**Table 3:** Genotype calling at the HLA locus from DNA-sequencing.

HLA gene name	Allele 1	Allele 2
A	HLA-A*02:01:01	-
B	HLA-B*15:11:01	-
C	HLA-C*03:03:01	-
DRB1	HLA-DRB1*01:01:01	HLA-DRB1*15:01:01
DQA1	HLA-DQA1*01:01:01	HLA-DQA1*01:02:01
DQB1	HLA-DQB1*05:01:01	HLA-DQB1*06:02:01
DPA1	HLA-DPA1*01:03:01	HLA-DPA1*02:02:02
DPB1	HLA-DPB1*04:02:01	HLA-DPB1*02:01:02
DMA	HLA-DMA*01:01:01	-
DMB	HLA-DMB*01:01:01	HLA-DMB*01:03:01
DOA	HLA-DOA*01:01:02	HLA-DOA*01:01:01
DOB	HLA-DOB*01:01:01	-
DRA	HLA-DRA*01:01:01	-
DRB2	Not typed	Not typed
DRB3	Not typed	Not typed
DRB4	Not typed	Not typed
DRB5	HLA-DRB5*01:01:01	-
DRB6	HLA-DRB6*01:01	HLA-DRB6*02:01
DRB7	Not typed	Not typed
DRB8	Not typed	Not typed
DRB9	HLA-DRB9*01:01	-
E	HLA-E*01:03:02	-
F	HLA-F*01:01:01	HLA-F*01:01:03
G	HLA-G*01:01:01	HLA-G*01:01:06
H	HLA-H*01:01:01	-
J	HLA-J*01:01:01	-
K	HLA-K*01:02	-
L	HLA-L*01:01:01	-
V	HLA-V*01:01:01	-

In conclusion, the designed array efficiently captured, amongst others, the *HLA-DR1* and *HLA-DR15* haplotypes characterizing THP-1 cells and combined with the HLA-HD tool, allows for up to 6-digit resolution of HLA haplotypes.

### Efficient and reproducible capture of designed regions with BS- and EM-seq

Genomic DNA samples derived from cells stimulated with either IFNγ or IFNγ/LPS were simultaneously assessed for DNA methylation after BS or EM conversion (**Figure 1C**), both methods resulting in the conversion of unmethylated cytosines to thymines, with methylated cytosines left unchanged.

**Figure 3:**
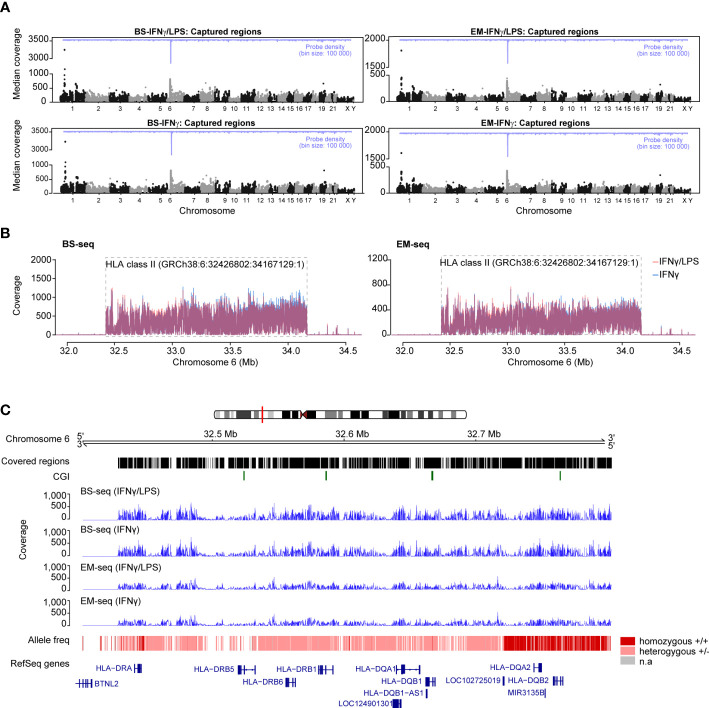
Capture efficiency and variant calling of the Bisulfite (BS) and Enzymatically (EM) converted libraries. **(A)** Manhattan plots of the median coverage in the BS-seq libraries (left panels) and EM-seq libraries (right panels) for the THP-1 cells treated with IFNγ/LPS (top panels) and IFNγ (bottom panels). The probe density is depicted across different chromosomes in blue. **(B)** Mean read coverage across the HLA class II locus on chromosome 6 (GRCh38:6: 32,426,802:34,167,129:1) for the BS-seq and EM-seq libraries. **(C)** Estimation of the allele frequency from the BS- and EM-seq libraries in the HLA class II region. The homozygous alleles are depicted with dark red and the heterozygous alleles with light pink. Regions that were of low coverage are illustrated by gaps. Mean coverage at the HLA class II locus for the BS- and EM-seq is depicted in blue. The RefSeq genes are depicted in blue.

Quality and adapter filtered reads were mapped against an *in silico* converted human reference genome (hg38), which accounts for C>T conversion at non-methylated sites. The total number of reads differed slightly between conversion methods but not between conditions (**Supplementary Table 4**). Accordingly, after deduplication, ~85% of the BS-derived and ~77% of the EM-derived reads mapped uniquely to hg38 reference (**Supplementary Table 4**), suggesting that the SureSelect protocol performs slightly better with downstream analysis using BS compared to EM-based conversion. The difference was less pronounced in the extended HLA class II region (GRCh38, chr6:32,426,802-34,167,129:1), where 90.0% (825) and 87.7% (804) of the 917 designed regions were captured by each method, respectively. The median coverage across BS-derived libraries was on average 91.5X (range 11X - 3263X) and reached 51X (range 11X - 1851X) across EM-derived libraries (**Table 4**). A higher coverage was generally estimated in the extended HLA class II region with an averaged median coverage for BS-seq of 151X (range 11X - 742X) and of 110X (range 11X - 446X) for EM-seq. Of note, all coverage estimations were conducted based on strand-merged CpGs and after applying a filter of 10X. The difference in sequencing depth and fraction of uniquely mapped reads between BS- and EM-derived libraries is further reflected in the number of designed regions that were captured for the DNA methylation libraries with an average of 89.8% (4,313/4,805) for BS-seq and 82.0% (3,941/4,805) for EM-seq, respectively (**Table 4**). The median coverage was also visually assessed with Manhattan plots across chromosomes, which showed an expected peak at chromosome 6 and consistency across libraries (**Figure 3A**). The peak on chromosome 6 was specific to the targeted class II region (GRCh38, chr6:32426802-34167129:1) and drastically decreased in up- and downstream regions (**Figure 3B**).

**Table 4:** Coverage of the regions captured using BS- and EM-sequencing.

	BS-seq		EM-seq		
	IFNγ/LPS	IFNγ	IFNγ/LPS	IFNγ	
All regions (n = 4,805)					
n regions (%)	4,298 (89.4%)	4,328 (90.0%)	3,970 (82.6%)	3,911 (81.4%)
Median coverage (range)	91 (11-3265)	92 (11-3261)	52 (11-1928)	50 (11-1773)
HLA class II locus (n = 917)					
n regions (%)	819 (89.3%)	832 (90.7%)	803 (87.6%)	805 (87.8%)
Median coverage (range)	149 (11-743)	153 (11-741)	110 (11-460)	110 (11–432)	

n, number; %, percentage.

We next asked whether DNA sequences from the BS- and EM-seq methylation data could be used for genotype calling using bcftools. The analysis yielded identical results compared to DNA-sequencing data, with *HLA-DRB1*,-*DRB6*, -*DQA1* and -*DQB1* loci being heterozygous, while homozygosity characterized *HLA-DQA2*, -*DQB2* and *HLA-DRA* loci, based on the bcftools-derived variants (**Figure 3C**).

In conclusion, the designed regions are also enriched with BS and EM conversion, this enrichment is reproducible between technical replicates, and ensures sufficient coverage for estimation of DNA methylation levels.

### Comparison of the detected CpG methylation between the BS- and EM-seq

**Figure 4:**
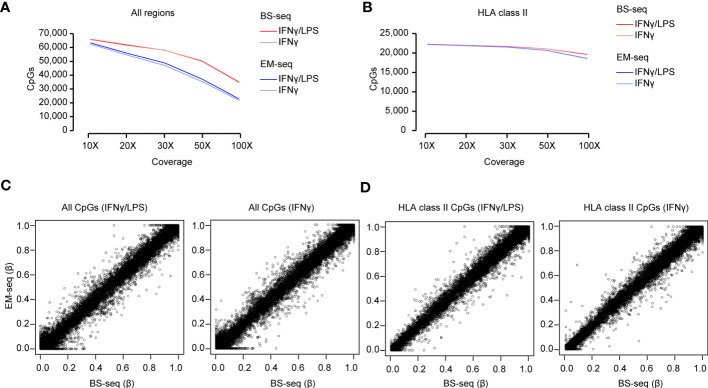
Methylation analysis of the Bisulfite (BS) and Enzymatically (EM) converted libraries. **(A)** Total number of CpGs captured with different coverage (10X, 20X, 30X, 50X and 100X) in the BS- and EM-seq libraries from THP-1 cells treated with IFNγ/LPS and IFNγ. **(B)** Total number of CpGs detected in the HLA class II locus with different coverage (10X, 20X, 30X, 50X and 100X) in the BS- and EM-seq libraries from THP-1 cells treated with IFNγ/LPS and IFNγ. **(C)** Correlation of CpG methylation (β-value) of the BS- vs EM-seq libraries for all the detected CpGs in THP-1 cells treated with IFNγ/LPS and IFNγ. **(D)** Correlation of CpG methylation (β-value) of the BS- vs EM-seq libraries for the detected CpGs in the HLA class II locus in THP-1 cells treated with IFNγ/LPS and IFNγ.

As a first step to accurately estimate DNA methylation, we assessed the coverage at individual CpG sites within the captured regions. The higher the coverage, the more reliable the estimation. An average of 65,908 and 62,813 CpG sites were covered with > 10X within the designed regions of BS- and EM-seq libraries, respectively, with approximately a third of them, i.e. 33.8%, 22,264/65,908 for BS-seq and 35.3% 22,179/62,813 for EM-seq, mapping to the extended HLA class II region. The relatively large percentage of CpG sites located outside of the HLA class II region is likely due to the enrichment of promoters within our design, which are generally enriched in CpG sites compared to other genomic features (65). Importantly, the majority of the total CpG sites, 98.9% (64,772/65,501) for BS-seq and 97.8% (60,625/61,996) for EM-seq, overlapped between conditions at 10X coverage, which allows for assessing changes in DNA methylation across samples. As expected, the number of CpG sites dropped with an increasing coverage threshold, resulting to an average of 76% and 57% (> 50X) and 53% and 36% (> 100X) of initial CpGs found using BS-seq and EM-seq, respectively (**Figure 4A**; **Supplementary Table 5**). This was likely affecting non-HLA regions since, in contrast, the number of CpGs at the HLA class II locus remained above 93% for both sequencings at 50X and as high as 88% and 84% for BS-seq and EM-seq, respectively, at a 100X coverage depth (**Figure 4B**; **Supplementary Table 5**).

We then compared the conversion efficiency of the two methods. DNA methylation levels were estimated as the number of Cs divided by the total number of sites. Apart from neurons and stem cells, DNA methylation generally occurs in a CpG context. Hence, non-CpG sites are commonly used for estimating conversion rates. The analysis of conversion efficiency showed that EM-seq seemed to perform better as only 0.6% of total non-CpGs were methylated, compared to an average of 1.5% for BS-derived samples (**Supplementary Table 4**).

In the next step, we compared the methylation levels detected by each method in the same samples, i.e. cells treated with either IFNγLPS or IFNγ at 10X coverage. We observed a linear correlation between methylation levels at all CpGs between EM-seq and BS-seq at all CpGs (Spearman r = 0.79, for IFNγ/LPS, r = 0.79 for IFNγ) as well as CpG sites located in the HLA class II locus (Spearman r = 0.93 for IFNγ/LPS, r = 0.94 for IFNγ) (**Figure 4C**).

### Functional annotation using differentially methylated CpGs

**Figure 5:**
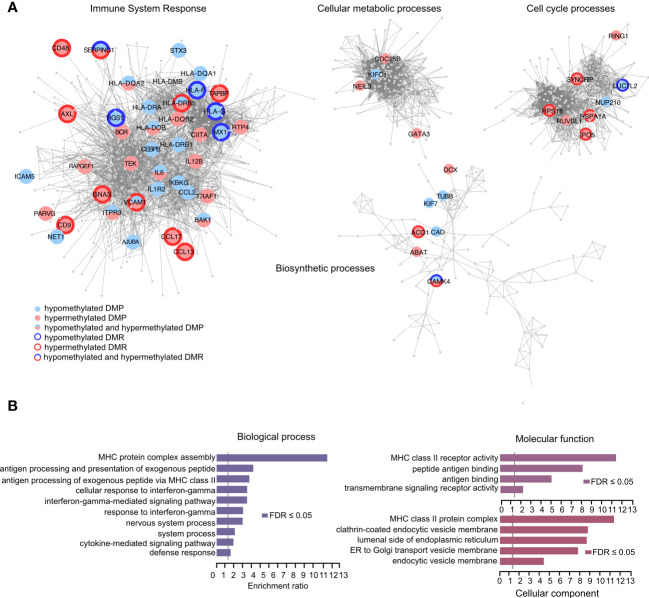
Identification of biological processes and molecular functions using the methylation capture design. **(A)** Graphical representation of the genes that associate with differentially methylated positions (DMPs) between THP-1 cells treated with IFNg/LPS and IFNg. Hypermethylated and hypomethylated positions are depicted in red and blue, respectively, and genes that displayed multiple, often consecutive, DMPs, referred to as differentially methylated regions (DMRs), are outlined in bold. Network and Gene Ontology (GO) analysis using STRING and Cytoscape were used as a backbone to demonstrate functions of the genes that were selected for the capture design. **(B)** Over Representation Analysis of GO terms in WebGestalt shows biological processes and molecular functions that associate with DMP-associated genes.

We next functionally explored the differentially methylated positions (DMPs) identified in the samples exposed to IFNγ alone or in combination with LPS. We focused on the DMPs that displayed > 10% difference in one method and at least 5% in another with the same directionality in both methods for methylation analysis (BS- and EM-seq). We performed STRING pathway analysis followed by community clustering to subdivide the number of pathways and graphically illustrate the differential methylation observed in core genes of each network (**Figure 5A**). In total, 329 DMPs mapping either promoters or enhancers in close proximity of promoter/enhancers of 150 genes (**Supplementary Table 6**) were selected for the analysis and segregated in the different pathways (**Figure 5A**). From these genes, some displayed hypomethylated and hypermethylated DMPs with or without hypomethylated/hypermethylated DMRs. In a next step we performed GO analysis of the differentially methylated genes and found significant (FDR < 0.05) enrichment of ‘biological processes’ related to *MHC complex assembly*, *antigen processing and presentation via the MHC class II* and *antigen processing and presentation of exogenous peptide antigen pathways* (**Figure 5B**). Moreover, we detected significant enrichment of the altered genes in the *interferon-gamma mediated signaling pathway* and *response to interferon-gamma*. Consistent with this, terms related to ‘molecular functions’ showed enrichment for the *MHC class II receptor activity*, *peptide antigen binding and antigen binding* (**Figure 5B**). GO categories linked to ‘cellular component’ highlighted enrichment in the MHC class II protein complex as well as pathways involved in ER and Golgi transportation and endosomal processing (**Figure 5B**).

These data demonstrate that with the current design we can simultaneously interrogate both the HLA regulation as well as the functional state of the myeloid immune lineage.

## Discussion

We developed a custom-made probe-based approach to simultaneously decipher the genetic and epigenetic variation in the HLA class II locus and more than 2,000 immune-related promoters. DNA-, BS- and EM-sequencing in stimulated monocytic THP-1 cells confirmed the robustness and efficiency of the capture of the targeted regions with high coverage. Moreover, we could successfully impute the HLA alleles with 6-digit resolution. Both BS- and EM-sequencing enabled quantification of CpG methylation across the HLA locus and the targeted promoters with high confidence, while promoter methylation status across immune genes additionally aided in the assessment of the cellular functional state. Thus, this methodology has the potential to unravel the intricate interplay between genetic and epigenetic regulation, particularly at the notoriously complex HLA locus, and to potentially provide novel insight into the pathogenic mechanisms underlying immune diseases.

The methodology developed in our study allows for deep sequencing of the HLA class II locus and HLA typing at a 6-digit resolution. This further provides a reliable avenue to deconvolute the complex genomic architecture of the locus, displaying highly polymorphic, multiallelic and haplotypic structure. Indeed, despite a massive success in sequencing the human genome across populations (66, 67) and annotating genetic elements and variation with regulation of gene expression in a tissue-specific manner (43, 68, 69), the characterization of the HLA locus remains unresolved. Throughout the years, several approaches have been used for the typing of the HLA locus, including PCR- and capture-based approaches. PCR-based methods can often result in allele drop-out due to amplification errors. On the contrary, capture-based approaches yielding longer sequences allow thorough paralleled investigation of multiple alleles in comparison to the PCR-based approaches. Several exome sequencing and whole genome sequencing kits have been developed for HLA typing and used in several projects (70). However, phasing of the haplotypes that involves delineation of the individual sequence strands is often of critical importance, as this process will delineate not only the allelic composition of each haplotype, but also important intergenic regions that exert regulatory roles. This enables dissecting the full genetic variation and interrogate the potential SNPs that could have an impact on diseases. Moreover, the phased haplotypes can be used as good annotation tools for variant calling and comparative studies (71).

Sequencing of the HLA locus requires high coverage for accurate variation calling and imputation of the alleles. Although, whole genome sequencing and whole exome sequencing have been used for the HLA, they are still relatively costly. Thus, a more targeted approach can give better coverage with a considerably reduced sequencing. The first RNA probe-based targeted capture of the HLA locus, also utilizing Agilent SureSelect^XT^ design, was developed against all reference alleles from the HLA database, in total 8,159 alleles for the eight classical genes of the HLA locus (72). The design included 4-time less probes (16,351 versus 65,384 probes) in comparison to the HLA class II locus alone in our design, with the same probe size of 120bp in both methods. It is noteworthy that this method relies on non-overlapping probes, as opposed to our 3X probe tiling, reducing the confidence of the calling. While this design, applied to 357 different cell lines, showed 700-fold enrichment of the HLA region, alignment revealed only 5% perfect read mapping to the reference sequences, which is substantially less compared to 98.6% in our method. Additionally, this method does not allow for distinguishing novel alleles as the design was based on the annotated alleles (72). We demonstrated that both HLA haplotypes were equally well captured using our protocol, although assessment of additional haplotypes is needed in the future. Another HLA-targeted approach exploited a probe set composed of a mixture of 80-bp oligonucleotides designed to recognize alleles from eight different homozygous cell lines. Approximately 99.6% of the HLA locus could be mapped with more than 10X. This number is similar to our mapping coverage (99.7%) with > 10X coverage for the HLA region. A more recent technique, based on the CRISPR system combined with long read sequencing, has been used for the dissection of the whole HLA region from one cell line (73). The advantage of this methodology lies on the accuracy of the haplotype calling, with large deletions being detected. However, this method is computationally demanding and challenging for large-scale projects as it requires extensive sample preparation. Whole genome and exome sequencing approaches used to reconstruct the haplotypes of individuals, such as in the SweHLA study, suggested that repetitive elements impair the read quality, with short read sequencing inducing biases in the imputation among similar alleles and genes, thereby influencing the reconstruction of the haplotypes. This was particularly the case at the *HLA-DRB1* gene locus, with repetitive elements displaying up to > 70X coverage in the exon 1, 3 and 6 of the gene (74). Our approach which was based on the masking of the repetitive elements provides more favorable outcomes in terms of read alignment to the reference genome and elimination of reads. However, we did not mask the pseudogenes in the target region thereby providing the possibility to examine their potential biological role. Pseudogenes, including those in the HLA class II locus, have typically acquired many mutations, insertions and deletions, which allows them to be distinguished from the original genes and analyzed using bioinformatics approaches. Indeed, we could confidently type DRB6*01:01, DRB6*02:01 and DRB9*01:01 present in the HLA-DR1/DR15 haplotypes. Nevertheless, we found an enrichment of the reads in a region on chromosome 1 that encompasses the following genes, *PPT1*, *EXO5*, *NFYC* and *RIMS3*. These genes have been found to be present in higher copy numbers (CNV standardized value 2.3) in the THP-1 cell line, as reported by the COSMIC database (75, 76). The higher coverage is therefore likely to result from structural variation in this locus in THP-1 cells specifically, the expression of these genes could be involved in proliferative ability and the leukemic status of these cells.

Apart from the HLA class II locus, our probe design included more than 2,000 immune-related promoters, selected based on their high relevance for immune functions in myeloid cells. We address potential changes in promoter methylation by comparing CpG methylation levels in THP-1 cells exposed to IFNγ alone or in combination with LPS. We focused on consistent methylation differences, i.e. with the same directionality in both BS- and EM-converted libraries in IFNγ compared to IFNγ/LPS at 323 differentially methylated CpGs mapping to 140 genes, 49 of them displaying regional changes at additional adjacent CpGs. A substantial number of differentially methylated positions was found in HLA class II genes (*HLA-DRB1, HLA-DRB5, HLA-DQA1, HLA-DQB1, HLA-DQB2, HLA-DPB2 and HLA-DRA*) involved in antigen presentation while the non-HLA affected genes were related to cell cycle and metabolic processes. Accordingly, overrepresentation analysis for the DMP-associated genes revealed significant enrichment of categories associated with HLA complex assembly, IFNγ signaling and vesicular endocytic pathways. These results concord with a previous study demonstrating the regulation of HLA genes together with costimulatory molecules upon IFNγ stimulation of THP-1 monocytes (77). Thus, the fact that our genetic and epigenetic targeted capture design combines comprehensive mapping of the HLA class II locus, including intergenic regulatory regions, with functionally relevant immune gene promoters, support the possibility to probe cellular states and dysfunctions. This unique feature is highly relevant given the significant enrichment of causal disease variants in such regulatory regions (78).

While the methodology developed in our study allows the simultaneous investigation of genetic and epigenetic variation in the HLA class II locus and more than 2,000 immune related promoters, there are certain limitations of the current protocol. First, comparison of BS- and EM-based methods to quantify methylation showed that BS performed better in terms of sequencing depth and outcome. This is surprising, considering the advantage of the EM method that preserves larger DNA fragments throughout the whole process (59). However, the original SureSelect protocol was optimized for BS-based conversion and further optimization of EM, such as changing the elution conditions, is warranted. Moreover, our design does not include the use of unique molecular identifiers (UMIs) tracing each molecule, which has been shown to facilitate the deduplication process and increase the accuracy of the sequencing both for DNA and RNA (79). As such, by applying this methodology to RNA-sequencing of peripheral blood cells, a previous study could decipher changes in the expression of the HLA alleles and distinguish allele-specific expression in the samples (80). Thus, future improvements can be implemented with the current custom-made probe library to further enhance a thorough characterization of the locus across different haplotypes.

Taken together, our methodology provides a technological advance for simultaneous characterization of the genetic and epigenetic variation in HLA class II region and for the complementary probing of proximal promoters of more than 2,000 immune-relevant genes. This provides the possibility to comprehensively assess the complex interplay between genetic and epigenetic regulation of HLA genes and further opens new horizon for the study of diseases that strongly associate with the HLA variation such as autoimmune diseases.

## Data availability statement

The data presented in the study are deposited in the Gene Omnibus Database (GEO, https://www.ncbi.nlm.nih.gov/geo/) repository, accession number GSE238121.

## Ethics statement

Ethical approval was not required for the studies on humans in accordance with the local legislation and institutional requirements because only commercially available established cell lines were used.

## Author contributions

MJ conceptualized and designed the study with input from MK and EE. MJ, LK and MN supervised the study. MK conducted all experimental procedures, network and enrichment analysis. CP assisted with library preparations. KH and SL designed the capture probes and SL conducted analysis of capture efficiency. MN performed majority of sequencing and efficiency analysis with input from MK and LK. MN performed haplotype calling with input from KS. MK, MJ, LK and MN wrote the manuscript. All authors read and approved the final manuscript.
