# Supplementary material for: Simultaneous detection of DNA variation and methylation at HLA class II locus and immune gene promoters using targeted SureSelect Methyl-Sequencing
Source: Front Immunol. 2023 Aug 24;14:1251772. doi: 10.3389/fimmu.2023.1251772 (PMC10484099; doi:10.3389/fimmu.2023.1251772)
Supplement: Supplementary file 2 [file DataSheet_1.docx]

**Supplementary Figures**

**Supplementary Figure 1**

**Supplementary Figure 1. (A)** Graphical illustration of different probe tilling densities to cover a specific region of the genome. **(B)** Probe boosting strategy for the improvement of the probe efficiency according to the GC context of the genome.

**Supplementary Figure 2**


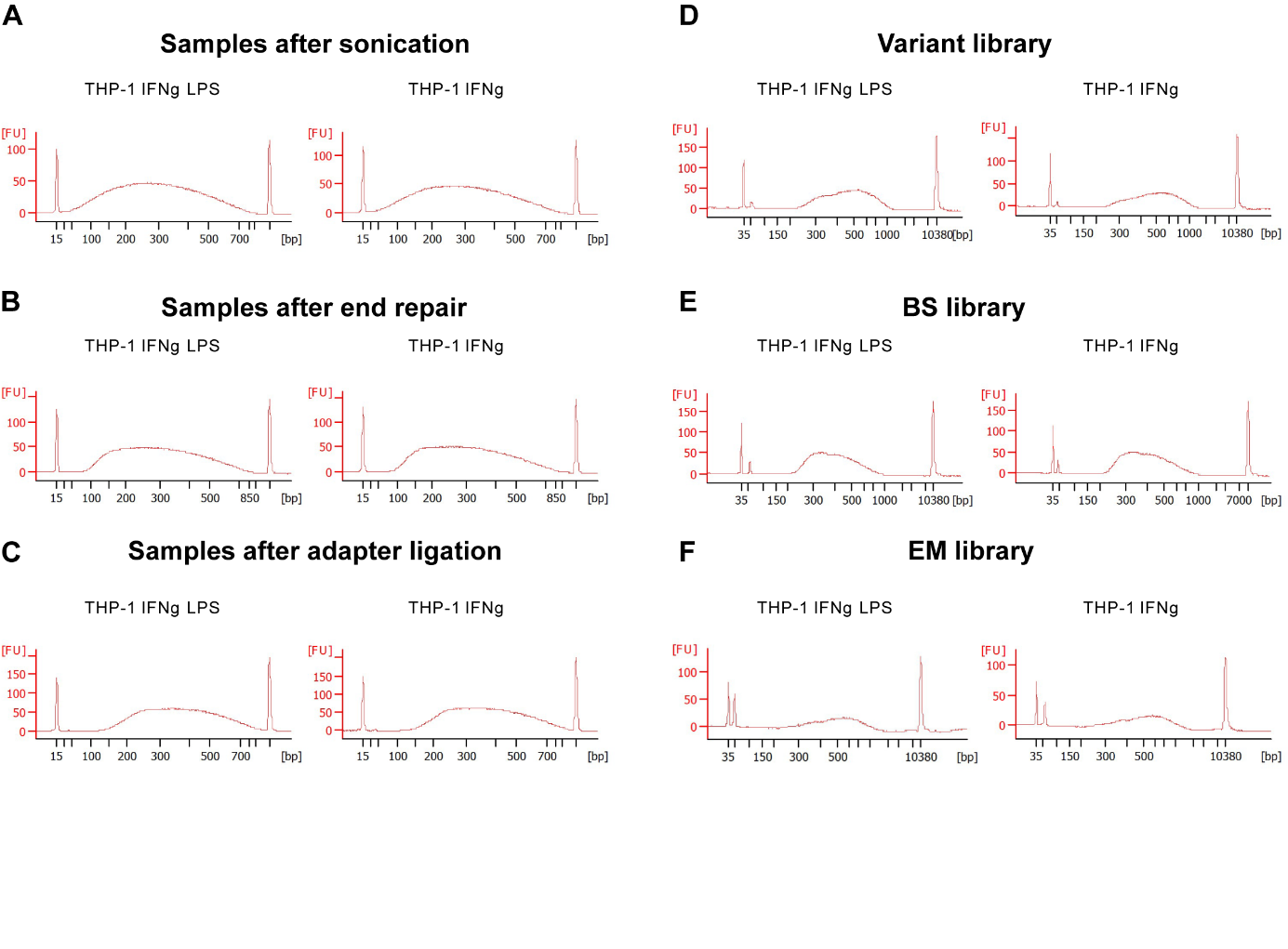


**Supplementary Figure 2.** Bioanalyzer profiles showing the average fragment size after sonication of the samples **(A)**, end-repair **(B)** and adapter ligation **(C)** for the THP-1 cells treated with IFNγ/LPS (left) and IFNγ (right). Final size of the Variant **(D)**, Bisulfite converted **(E)** and Enzymatically converted **(F)** libraries for the IFNγ/LPS (left) and IFNγ (right) treated samples.
